# Worldwide legislative challenges related to psychoactive drugs

**DOI:** 10.1186/s40199-017-0180-2

**Published:** 2017-06-02

**Authors:** Carolina Negrei, Bianca Galateanu, Miriana Stan, Cristian Balalau, Mircea Lucian Bogdan Dumitru, Eren Ozcagli, Concettina Fenga, Leda Kovatsi, Domniki Fragou, Aristidis Tsatsakis

**Affiliations:** 1 Departament of Toxicology, Faculty of Pharmacy, “Carol Davila” University of Medicine and Pharmacy, Bucharest, Romania; 2 Department of Biochemistry and Molecular Biology, University of Bucharest, 91-95 Spl. Independenței, 050095 Bucharest, Romania; 3 Department of Surgery, “Sf. Pantelimon” Emergency Clinical Hospital, Bucharest, Romania; 4 Bucharest Bar Association, Romanian Lawyers Union, Bucharest, Romania; 5 Department of Pharmaceutical Toxicology, Faculty of Pharmacy, Istanbul University, Beyazit, 34116 Istanbul, Turkey; 6 Department of Biomedical and Dental Sciences and Morphofunctional Imaging, Occupational Medicine Section, University of Messina, 98125 Messina, Italy; 7 Laboratory of Forensic Medicine and Toxicology, School of Medicine, Aristotle University of Thessaloniki, Thessaloniki, Greece; 8 University of Crete, Medical School, Department of Toxicology and Forensic Sciences, Heraklion, Greece

**Keywords:** “New” psychoactive substances, Regulatory, Control systems, Legislation

## Abstract

**Abstract:**

The discovery of a “new” psychoactive substance is a relatively exceptional event, while the regulatory response usually involved the assessment of risks to public health and inclusion of the novel substance in the national list of controlled substances. However, in recent years we have witnessed the rapid emergence of new chemical substances, which elude international control and pose a challenge to existing processes and a threat to the credibility of control systems. We currently review and present characteristics of these legal and illegal new substances and issues regarding their global monitoring and regulatory measures already taken, or in the process of being taken, for their control. The concept of prohibition applied in active substance-related legislation is rather hazard ridden as balance is required between the ban on substances of potential therapeutic use and the access on the market of high-risk substances.

**Graphical Abstract:**

Current and future laws regarding psychoactive compounds.
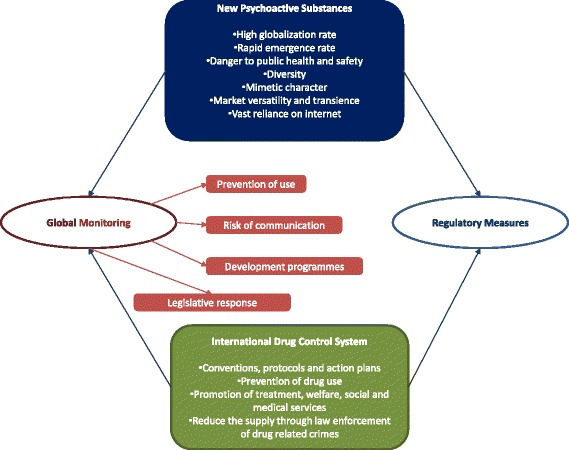

## Background

The World Health Organisation (WHO) has defined psychoactive substances as substances that, when taken in, or administered into one’s system, affect mental processes such as cognition. The term “psychoactive substances” comprises both legal and illegal substances. The WHO is committed to assisting countries in the development, organisation, monitoring and evaluation of treatment and other services in order to reduce the burden of psychoactive substance use. Today, we acknowledge the continuously growing threat of the emergence of new psychoactive substances, also known as “legal highs”, “designer drugs” or “herbal highs”, which are mainly developed for recreational use. These substances require worldwide cooperation in order to ensure evidence-based, multidisciplinary and integrated response [1–8]. In some cases, these substances are even sold with the notification “not for human consumption”, in order to bypass existing laws.

The international drug control system mainly relies on integrated and generally accepted strategies, which include conventions, protocols and action plans (for material reference, please see the indicated timelines as provided in various international documents) [9]. These strategies evolve in order to respond to scientific and general progress.

Drug-related policies and law enforcement actions rely on inter-state collaboration, since it is a manifest problem and responsibility of all countries. The body of policies, strategies and action plans in the controlled substances domain should be developed taking into account local, regional and international socioeconomic issues as well as requirements of alternative, sustainable development, as “complementary and mutually reinforcing” [10].

In the past decade, the world has become increasingly vulnerable to a novel danger, namely the emergence, at a record rate, of the so-called “new” psychoactive substances. In line with international provisions, mainly initiated by the United Nations, as well as other, regional, specialised bodies [11], such psychoactive substances are not new in terms of their recent development, but in terms of the novelty of their use for their psychoactive effect [12].

This newly defined category of substances may include any single substance/preparation not listed in the tables provided by the UN Single Convention on Narcotic Drugs of 1961. Nevertheless, they present with a health harm potential similar to that of chemicals customarily listed in Schedule I, II or IV of the Convention.

Many regulatory authorities, such as the European Medicines Agency, are working on enhancement of psychoactive and abused medicines monitoring. The laws regarding new psychoactive substances are quite complex and differ from one agency to another. However, these laws generally become stricter in time all around the world due to health risks. Especially across United Kingdom, new laws introduce a blanket ban on the “new” psychoactive substances effecting their sale, production, distribution and supply. The main aim of the UK Psychoactive Substances Act 2016**,** for instance, is to resolve such shortcomings by banning all psychoactive substances, which are now considered illegal, except for food, alcohol, nicotine, caffeine and medicinal products.

Similar in effects to “classic” drugs, the “new” psychoactive substances differ from the former in the fact that they lack any present or past medicinal use. The main purpose of their wide scale production and trade is circumvention of regulatory restrictions, devised for the control of their “traditional” counterparts. Another significant difference would also be increasing prevalence of internet networks for their sale, a very different approach compared to conventional illegal markets.

Perhaps the most important characteristic of the “new” psychoactive substances is their unparalleled *globalisation rate*. By December 2015, the UNODC (The United Nations Office on Drugs and Crime) had reported the presence of over 643 “new” psychoactive substances in 101 countries. More substances have been reported in Europe than any other region. According to a study conducted in China, Japan, Korea and Taiwan, between 2007 and 2015, among a total of 940 “new” psychoactive substances reported, only 25 were at the time controlled in four countries, with Japan and Korea being the most proactive in regulation. Another alarming characteristic is their extremely *rapid emergence rate*. In 1900, the “new” substances reported had been just marijuana and mescaline; by 1950, 20 more had already been reported, followed by 280 in 2000 and over 600 reported to the UNODC by December 2015. Furthermore, the “new” psychoactive substances show diversity of *danger to public health and safety*. Harmful effects range from various degrees of poisoning, going through the high potential for acquisition and transmission of HIV and other haemo-communicable diseases when administered intravenously, triggering of cardiac conditions and seizures, psychotic manifestations, lethal outcomes included, to traffic events caused by driving under the influence. Additional risks in that respect are related to intentionally misleading labelling and packaging, resulting in user ignorance about the substance exact composition and degree of purity, thus increasing the risk of overdose.

Moreover, new psychoactive substances are *diverse* in terms both of effects and chemical structure; they exhibit a *mimetic character* (i.e. effects of “new” psychoactive substances identified so far imitate those of the major groups of controlled substances); they have a *mixed character* (i.e. absence of specific emergence patterns worldwide). Other characteristics include a well-defined tendency for sale as *“mixes”* with other substances that the user may or may not be aware of, or cognizant with, bearing direct impact on risks; distinct *market versatility*, with prompt responsiveness to changes in legal controls and transfer among markets upon emergence of stricter legislation; and, last but not least, definite *market transience* as well as vast reliance on the *Internet* for advertising and sale [13–17].

Among the purposes of the international drug control system, one should mention prevention of drug use through the decrease of demand and other such measures, as well as promotion of treatment, welfare, social and medical services for drug-related conditions. Last but not least, one should not overlook reducing the supply by consistent, proportionate and effective law enforcement against drug-related crime.

Current developments in chemistry and technology support development of new psychoactive substances, some of which are much more potent compared to the model drugs they are designed to replace. It is also an acknowledged fact that detection of these substances in biological samples involves certain difficulties [5]. Several analytical techniques have been developed and different biological samples were also assessed in order to overcome these difficulties [18–20]. The fact that the pharmacological and toxicological effects of these substances are not fully defined and their rapid emergence on/disappearance from the market are the most important challenge for management of related risks.

Over the years, emergence of such “new”, potentially harmful psychoactive substances eluding international control has resulted in increased substance abuse and related adverse effects (overdose, hospitalisation and lethal outcomes). Prediction of abuse liability for new psychoactive substance has a key role in their safety, however hundred percent accuracy is not feasible for many cases. As existing pharmacovigilance systems are a solution for tracking undesirable effects of psychoactive drugs, active safety monitoring of illegal psychoactive substance is a problem that has not yet been solved.

## Contributing factors

Among factors acting in favour of the increasingly extensive use of this drug category are the lack of appropriate regulations, the rapid development and shorter time to launch on the market and therefore the lower price, resulting in higher availability and accessibility, particularly for young people, as well as the speed, unpredictability and localisation of marketing patterns.

The tremendous current proliferation of “new” psychoactive substances, this combination of diversity and rapid emergence has markedly complicated the global trend in drug abuse and its regulation is becoming a matter of worldwide concern.

The obvious and very forceful paradigm shift in drug use, towards prevalent use of synthetic drugs, the number, diversity and incompletely clarified market patterns pose a novel and major challenge to international policy makers, exerting significant pressure on systems for the development of national policies and response strategies in the field, requiring major rethinking and reorganisation of traditional political, legal and operational approaches and tools.

## Global monitoring

Considering all particularities, the effort to *manage* the complex issue of “new” psychoactive substances requires several lines of approach and intervention, the results of which are essential to provide an evidence base for regulatory decisions in this area [21–23].

An important issue is their systematic and global monitoring. “New” psychoactive substances detection and identification are a first essential step in the development of strategies for reduction of supply and design and implementation of therapeutic interventions, as well as for collection of accurate data for effective policies. In that respect, insufficient identification, analysis and reporting have become a known factor for poor information and regulation [24]. Pharmacovigilance activities have key role in maintaining safe usage of drugs. Risk management plans related to psychoactive drugs may minimize possible risks and these plans should also include legal, social and patient related concerns [25].

Policies, strategies and action plans, as well as early warning networks and implementation rules therefore have to address not only the harmful health and social effects of such substances (by preparing suitable national models for prevention, control and treatment), but also to provide scientific evidence for analyses and propose scheduling schemes for the most widespread, persistent and harmful “new” synthetic substances.

A second dimension of this global approach is the concern for prevention of use and deterrence of misuse and diversion of medically used psychoactive and psychotropic substances, whilst at the same time preserving their availability for medical, legal purposes, which has been transferred from the approach of “traditional” drugs to “new” ones.

Along the lines of risk communication, significant importance is assigned to establishing, strengthening and actively participating in information and good practice exchange within networks (early warning and other) as well as in cross-partnerships among policy makers, authorities, law enforcers, the chemical and pharmaceutical industries and researchers across various fields (health-related, chemical, pharmaceutical, social and welfare, educational etc.).

Of special importance are approaches meant to offset the drug-related use of the internet, as one of the most difficult aspects of control and a major factor contributing to the globalisation, speed and unpredictability of the market for “new” substances. Good pharmacovigilance activities as well as risk management plans promise to enhance rational usage of these drugs. The Internet should be used for prevention purposes, for provision of suitable counselling and information, using the social media and other social networks for implementation and promotion of the various strategies and actions, preventing involvement of potential users, particularly children and young people, in illicit drug sale and purchases [26, 27].

On a more general scale, strategies for approaching the “new” psychoactive substances issue have to fit into frames of long-term and sustainable development programmes, aimed to address such drug use-generating socioeconomic effects as unemployment and social marginalisation [28].

An additional but perhaps the most important dimension of national and international reaction to the hazards imposed by “new” psychoactive substances is legislative response [29].

In the atypical and challenging context of the “new” psychoactive substances, a wide range of regulatory responses have been explored in the joint effort towards protection of public health, to adjustment to the specific market dynamics, characterised in particular by the speed of their emergence and their diversity, the manufacturers’ relentless attempts to elude the law and the limited nature of data required for full assessment of harm.

For a better description of the intricacies of the specific market for “new” psychoactive substances, one must not overlook that the placing under legal control of potentially harmful substances can be a lengthy process, requiring prior thorough collection of specific information, followed by scientific assessment and a consultation process. An inevitable, significant gap thus occurs between the timing of a new substance emergence and application of the respective legal control, used to the advantage of developers and traders of a novel synthetic substance, which opens the possibility to circumvent controls.

In addition, prompt scientific assessment after emergence of a “new” substance on the market is difficult and collection of a sufficient amount of data necessary for regulatory decision requires time, which is not available to legislators.

Having such intricacies in mind, prompted by the need for faster response, the options currently available are the use of interim and discretionary measures within the limits of international conventions, for emergency purposes and prevention of larger scale use before establishment of proper international control and setting priorities for regulation of “new” psychoactive substances, allowing for focus on the most persistent, prevalent and harmful ones [30].

However, the customary dilemmas associated with regulatory decision-making are further complicated by lack of/limited information required for evidence-based, scientific [31] and adapted response, on issues such as the possible harmful effects on users, the substance potentially used as complement to/substitute of psychoactive substances of demonstrated higher risk and the possible harmful effects of illicit markets resulting from prohibition.

Currently, in direct relation to the diversity of “new” psychoactive substances, there is a great variety of legislative options for national control, as adopted by the various countries. Such options can vary in line with the number of “new” psychoactive substances identified nationally, and may include the use of individual lists, of analogic/generic patterns of control, transfer of consumer protection/medicinal product regulatory provisions [32, 33], seizures, use of specific legislation (ranging from general prohibition of distribution to institution of pre-licensing regimes) and strengthening laboratory detection and identification capacities [34, 35].

Given the scale of negative consequences for policy makers resulting from marketing authorisation of substances to be proved dangerous later on, there is a probably inherent and therefore unavoidable tendency of systems to ban “new” substances about which insufficient information is known. So far, authorities have been limited largely to regulatory option referring “new” psychoactive substances to the framework adopted for psychoactive substances with controlled regime [36].

By comparison, the negative consequences of the decision to hold off the market substances that are actually harmless or maybe even useful as substitutes of psychoactive substances of recognised harmful potential, even at the cost of aggravating problems resulting from the ban, are minimum.

This explains the option to prohibit these “new” substances about which there is too little information, as “a precaution”.

In recent years, quite a lot of attention has been paid to this “principle of precaution”, mainly taken into account in policy-making under uncertainty. In the context of the “new” psychoactive substances however, things are complicated by the fact that taking account of the potential harm only leads to ignoring the more elusive, but actual dangers arising from the ban decision. Currently, no or very little account is taken of any of the potential benefits justifying the individual’s decision for use. The dilemma is further complicated in that any legalisation may lead to unwanted consequences such as more extensive substance abuse [37].

Under the current legislative decision-making system, in relation to “new” psychoactive substances, a fundamental concern is achievement of a more balanced approach in decision-making, able to counteract the inherent propensity for automatic ban.

So far, the argument in favour of prohibition is undeniable and seems to have proved effective in the current system with regard to “new” psychoactive substances, apparently generally succeeding to avoid the major problems possibly arising from the decision to allow the marketing of substances that can possibly prove dangerous in time.

Therefore, achievement of a flexible and adapted approach is only possible through consistent research efforts to better understand the potential of new entities as substitutes of substances recognised as harmful and the harmful market effects resulting from banning use of these entities.

Thus, in addition to the slow process of legislative response, a further disadvantage of the ban option is looming specific to international and national approach of regulatory action, i.e. implicit encouragement of the decision to prohibit based on the context of uncertainty.

Underlying this almost general manner of approach, which is applied because of uncertainties arising from the limited amount of information, the precautionary principle can be defined in terms of the Rio Declaration on Environment and Development of 1992 i.e. “Where there are threats of serious or irreversible damage, lack of full scientific certainty shall not be used as a reason for postponing cost-effective measures to prevent environmental degradation” [38]. According to this phrasing, this is a very modern principle, which considers scientific knowledge as a key issue for policy, especially regulatory decisions.

Cases where application of provisions of this declaration was recommended involved circumstances requiring urgent measures to deter possible hazards to humans, plants, animals or the environment when existing scientific knowledge did not allow full risk assessment. As clearly stated, the principle cannot be used as a reason to enforce protective measures and is mainly applied in case of threats to public health (the same as, for example, in cases of withdrawal from the market of products suspected of posing health risks). That is why the phrase is quite suitable for making regulatory decisions with regard to “new” psychoactive substances, which are indeed characterised by uncertainty as to their true nature benefits and harms, as well as to their potential risk to public health.

## Regulatory measures

And yet, the current review of the context of regulation of psychoactive substance use sees application of this principle as a conservative attitude, given that, for all psychoactive substances considered so far, the dangers are not proven at the same extent for all users.

Regardless of such considerations, the principle states that the authorities’ duty is to protect individuals from such threats, seemingly favouring adoption of controlled regimes and inclusion of such substances in the existing schedules currently provided by international and national law.

Routine enforcement of precautionary measures in the control and management of “new” psychoactive substances is not devoid of disadvantages however. For instance, the penalties provided in the prohibition law may be more harmful than the substance itself: addition to the individual’s criminal record or imprisonment for possession is often more detrimental to the individual and the society than the substance itself because it limits the person’s opportunities and it generally changes the course of their life, especially for young people. In addition, one should not ignore the costs of legal proceedings and public cost for imprisonment. Adopting a cautious attitude and subsequently banning a wide range of illegal psychoactive substances leads to unlawful market development and competition that may prove harmful to society. At the same time, the precautionary principle does not take into account the proportionate drug-specific risks and absolute level of risk required to ban a drug. For example, there are very dangerous substances on the market (alcohol and tobacco, particularly) whose regulation is still in progress, while other substances of lesser harm to individuals and society are already prohibited.

In this direction, it has already been argued on the need to develop, as in the case of other risky behaviours such as climbing, for instance, indicators serving as risk thresholds, meant to be used as foundation for any decision to ban any psychoactive substances. Frequently, caution is based openly or implicitly on the argument related to the lack of benefits in drug use, in itself a justification for ban. Such an argument, however, reflects a biased and deeply rooted institutional position, i.e. authorities and regulatory decision-makers are the only ones able to understand the respective costs and benefits. This situation excludes user’s motivation (subjective user-perceived benefits, e.g. relaxation, enhanced creative capacities and perception etc.) from the equation of consumption, and ignores an undeniable reality. The most costly and twisted consequence of the precautionary principle in regulatory decision-making is encouraging the use of legal substances that are more harmful than the prohibited ones. At the same time, the precautionary principle limits the development of “new” substances safer than alcohol, for example. Moreover, one should not ignore the existence of certain psychoactive substances such as LSD (D-Lysergic Acid Diethylamide), MDMA (3,4-Methylenedioxymethamphetamine) and psilocybin, which had started as therapeutic agents before ban. On their prohibition, investigations were duly stopped, only to be resumed 40 years later. In addition, it appears that the recent ban on mephedrone has resulted in hindering the development of new antidepressants and other treatments for obesity and narcolepsy, for example. Legal and regulatory complexities in this area, the likelihood of legislative changes and the real possibility of banning any newly discovered substance are important deterrents for the pharmaceutical industry.

## Conclusions

It is an undeniable fact that psychoactive substances pose various hazards for the user.

As far as prohibition is concerned, making decisions to ban “new” substances and to include them in schedules for controlled substances poses certain issues for consideration, among which one should highlight their harmfulness of use both to the individual and the society; their inherent risk; their spreading; their potential for addiction; their harmfulness generated by the nature of the market; the connection with organised criminal acts; the harmfulness and expenses of their banning (e.g. user criminal prosecution, prevention of use as substitutes of more dangerous substances, loss of opportunities for quality control/providing scientific data about the substance etc.) and the user perceived benefits (e.g. relaxation, medical, improved performance).

Hasty prohibition of a “new” substance may create unnecessary problems associated with illegal markets, at the same time leading to very difficult collection of data for more evidence-based decision-making. On the other hand, tardy prohibition may allow widespread use of substances of significant addiction and/or hazardous potential.

Such decisions should not be taken before carefully reviewing the possibility of substance use as a replacement for a more dangerous one, which has been prohibited as such [39].

## Abbreviations

LSD: D-Lysergic Acid Diethylamide
MDMA: 3,4-Methylenedioxymethamphetamine
UNODC: The United Nations Office on Drugs and Crime
WHO: World Health Organisation
